# Changes of Trigeminal Ganglion Neurons Innervating the Temporomandibular Joint in Chronic Pain Rat Model

**DOI:** 10.1155/2024/7015382

**Published:** 2024-09-13

**Authors:** Wen Liu, Henghua Jiang, Jin Ke, Xin Liu, Yaping Feng, Jinsong Hou, Xing Long

**Affiliations:** ^1^ Department of Oral and Maxillofacial Surgery Hospital of Stomatology Guanghua School of Stomatology Sun Yat-Sen University, Guangzhou, Guangdong, China; ^2^ State Key Laboratory of Oral and Maxillofacial Reconstruction and Regeneration Key Laboratory of Oral Biomedicine Ministry of Education Hubei Key Laboratory of Stomatology School and Hospital of Stomatology Wuhan University, Wuhan, Hubei, China

**Keywords:** animal model, calcium signaling, chronic pain, temporomandibular joint osteoarthritis, trigeminal ganglion

## Abstract

**Background:** Phenotype alterations of nociceptive neurons have been shown to be a key step in the pathogenesis of many pain-related diseases. However, it is unclear if the characteristic changes of temporomandibular joint (TMJ) primary afferent neurons are related to the pathogenesis of temporomandibular joint osteoarthritis (TMJOA) chronic pain. This study aimed to determine the morphological and neurochemical changes in trigeminal ganglion (TG) neurons innervating the TMJ in TMJOA chronic pain rats.

**Materials and Methods:** Monosodium iodoacetate (MIA)-induced TMJOA chronic pain rat model was established (*n* = 6), and saline was injected in rats of the control group (*n* = 6). TMJ primary afferent neurons were labeled with retrograde tracing (Dil). The spatial distribution and the expression of calcitonin gene-related peptide (CGRP), isolectin B4 (IB4), and neurofilament 200 (NF200) of TMJ primary afferent neurons in TG were investigated using immunofluorescence. Intracellular calcium signaling was recorded by calcium imaging (*n* = 20).

**Results:** TMJ primary afferent neurons were located only in the V3 region of the TG from both saline- and MIA-injected rats. The number of TG neurons innervating the TMJ was increased in MIA-injected rats. Elevated number and intracellular calcium concentration of small- and medium-sized instead of large-sized Dil+ TG neurons were found in MIA-injected rats. The upregulated expression of CGRP and IB4, but not NF200, in TG neurons innervating the rat TMJs was accompanied by TMJOA chronic pain.

**Conclusion:** This study suggests that sensitization of small- to medium-sized Dil+ TG neurons and CGRP- and IB4-positive Dil+ TG neurons might contribute to the development of TMJOA chronic pain in rats. This will provide valuable information for more efficient control of TMJOA chronic pain.

## 1. Introduction

Temporomandibular joint osteoarthritis (TMJOA) is a major cause of temporomandibular disorder (TMD)-related chronic pain [1–3]. Chronic pain often drives TMJOA patients to seek medical care from dentists and severely impacts patients' quality of life [4]. There is no doubt that relief of pain is a top priority in the treatment of TMJOA. However, the basis for TMJOA-related chronic pain is not fully understood, which can impose a major challenge for clinicians [5]. Therefore, elucidating the pathogenesis of chronic pain in TMJOA is critical to the development of new, more efficient treatment strategies.

The trigeminal nerve provides the principal sensory innervation of the temporomandibular joint (TMJ) [6, 7]. Trigeminal ganglion (TG) neurons can be divided into three types: small (<30 *μ*m), medium (30–40 *μ*m), and large (> 40 *μ*m). Small- and medium-diameter neurons mainly transmit nociceptive information. In contrast, large neurons generally detect and transmit innocuous stimuli [8]. Orofacial noxious stimuli typically activate small- and medium-sized (C and A*δ* fibers) primary afferent neurons in TG. These nociceptive neurons conduct nociceptive information from their nociceptive nerve endings to the central nervous system [7, 9]. However, it remains unclear which type of TG neurons innervate the TMJ and whether they are involved in chronic pain from monosodium iodoacetate (MIA)-induced TMJOA.

In general, small- to medium-sized neurons express either calcitonin gene-related peptide (CGRP) or isolectin B4 (IB4), whereas large-sized neurons express neurofilament 200 (NF200) [10, 11]. Nociceptive stimuli excite nociceptors on the nerve endings of unmyelinated CGRP+ and IB4+ sensory neurons and evoke pain [11, 12]. However, recent studies have revealed that different neuronal populations are involved in pain hypersensitivity induced in different pain models by using the retrograde tracing method [9]. For example, in dorsal root ganglion (DRG) neurons innervating the mouse endplates, CGRP+ neurons but not IB4+ neurons are involved in spinal hypersensitivity induced by lumbar spine instability [13], while another study has shown that IB4+ primary afferent neurons play a significant role in carrageenan-induced inflammatory hypersensitivity [14, 15]. Based on our knowledge, the phenotype changes of TG neurons governing the TMJ in TMJOA rats experiencing chronic pain are not completely understood yet.

The present study was performed to clarify the morphological and neurochemical changes of nociceptive TG neurons innervating the TMJ in MIA-induced TMJOA rat. We first labeled primary afferent neurons innervating the TMJ within rat TGs by an intra-articular injection of retrograde tracer (Dil) and then determined the soma size and the expression of nociceptive markers (CGRP, IB4, and NF200) of Dil-labeled TG neurons.

## 2. Materials and Methods

### 2.1. Animals

In this research article, a total of 32 male Sprague–Dawley rats (250–300 g) purchased from the Experimental Animal Center of Hubei Province were used in this experiment. In this study, all animal procedures were approved by the Animal Research Ethics Committee, Hospital of Stomatology, Wuhan University, China (approval number: S07918120A), and were performed according to the guidelines of the International Association for the Study of Pain [16]. Rats (three to four per cage) were housed in a temperature-controlled room (23 ± 1°C, 12-h light–dark cycle) with free access to food and water.

### 2.2. Rat Models of TMJOA Chronic Pain

The TMJOA chronic pain was induced according to the previous study [17]. In brief, rats were randomly divided into MIA-injected group (*n* = 6 rats) and saline-injected group (control; *n* = 6 rats). After anesthesia with 1.5% isoflurane (Abbott Laboratories, North Chicago, IL, USA), the MIA (50 µl, 1.0 mg/50 µl; Sigma–Aldrich, St. Louis, MO, USA; Catalog: I2512) or saline (50 µl) was injected bilaterally and slowly into the upper compartment of the TMJ with a 27-gauge 0.5-inch needle. To minimize the suffering of the animals, Day 14 was selected to evaluate the phenotype alterations of neurons, as our previous work revealed that chronic pain started at Day 14 in the MIA-induced TMJOA rats [17].

### 2.3. Hematoxylin and Eosin (HE) Staining

After appropriate survival times, rats were deeply anesthetized with pentobarbital and perfused transcardially with ice-cold phosphate buffered saline (PBS) and 4% paraformaldehyde. After the perfusion, the TGs were dissected, postfixed (4°C for 12 h), and cryoprotected in 30% sucrose solution overnight. The 10-*μ*m sections of TG tissue were cut in a cryostat. Subsequently, to investigate the spatial distribution of TMJ primary afferent neurons in rat TG, we obtained images of the entire ganglion in HE-stained longitudinal sections of the TG using a Pannoramic digital slide scanner to observe each subregion of the TG.

### 2.4. Retrograde Tracing

By modifying methods described previously [18], we anesthetized rats on Day 9 after injection of MIA or saline. Then, 10 µl of Dil (Invitrogen, Catalog: D282, 2.5 mg/ml) was injected into the rat TMJs. Immediately after injection, the pinholes were sealed with sterile cotton to prevent reagent leakage. TGs were obtained for primary neuron culture or immunofluorescence at 5 days after Dil injection.

### 2.5. TG Neuron Culture

Neurons were enzymatically dissociated from TG of rats (saline-treated rats, *n* = 6; MIA-treated rats, *n* = 6) as described in the previous study [19]. Briefly, TG was first digested with collagenase D (2.5 mg/mL; Roche, Catalog: 11088866001) for 25 min (37°C) and then 0.25% trypsin (Gibco, Catalog: 25300120) for 10 min (37°C) after being cut into small pieces. Next, single neurons were isolated by gentle trituration using a sterile Pasteur pipette. After trituration, the isolated cell cultures were filtered through a 70-*μ*m cell strainer; the filtered TG neuron suspension was cultured on a pre-coated dish.

### 2.6. Calcium Imaging

The Fluo-3 AM (Shanghai Yeasen Biotechnology Co. Ltd., Shanghai) was used to measure calcium signal. Briefly, 2-mM stock solution of Fluo-3 AM was prepared in dimethyl sulfoxide. Then, the Fluo-3 AM solution was dispersed to final concentration at 4 *μ*M Fluo-3 AM. At 24 h after neuron culture (saline-treated rats, *n* = 4; MIA-treated rats, *n* = 4), the neurons were incubated with the corresponding reagents, and then the culture medium was removed, and the cells were washed with Hank's buffer for three times. Fluo-3 AM was added to a 24-well plate and incubated at 37°C for 20 min, and the cell loading medium was removed. After washing with Hank's buffer for three times, the cells were incubated with Hank's buffer for 30 min at 37°C and prepared for calcium imaging. The calcium imaging was captured using a fluorescence microscope (Olympus). Mean fluorescence intensity (MFI) and cell diameter were measured using Image-Pro Plus software (Media Cybernetics).

### 2.7. Immunofluorescence

The 2.5% bovine serum albumin was used to block nonspecific binding, and then the following primary antibodies were incubated overnight at 4°C: anti-CGRP (mouse, 1 : 1000, Abcam, Catalog: ab81887), IB4-FITC (1 : 200, Sigma, Catalog: L2895), or anti-NF200 (rabbit, 1 : 500, Novus, Catalog: NB300-135). Next, the slice was incubated in the relevant secondary antibody for 1 h at 37°C. Finally, 4',6-Diamidino-2-Phenylindole (DAPI) was used for cell nuclei staining. Images were obtained using a fluorescence microscope (Leica) and/or Pannoramic digital slide scanner (Leica). Image analysis was performed using Case Viewer software (Leica) and Image-Pro Plus software.

### 2.8. Statistical Analysis

In this study, data were analyzed using GraphPad Prism 8.0 (Insightful Science). Mann–Whitney's *U* test or two-tailed Student's *t* test was used in this study for comparisons between two groups, and *P* < 0.05 was considered to be significant.

## 3. Results

### 3.1. Localization of Primary Afferent Neurons Innervating Rat TMJs

**Figure 1:**
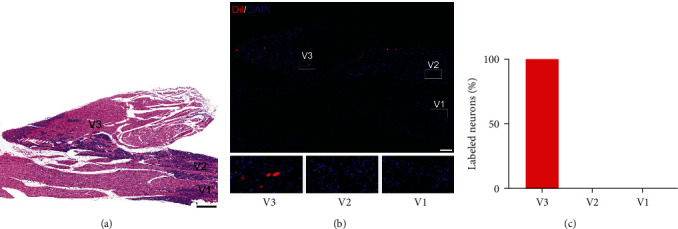
Spatial distribution of the TMJ primary afferent neurons in TG from untreated rats. (A) Representative images of HE-stained longitudinal sections of the entire TG. The TG consists of V1 region, V2 region, and V3 region; scale bars represent 200 *μ*m. (B) Representative images of DAPI-stained (blue) longitudinal section of the TG. A longitudinal section of the entire TG is shown in the upper panel; scale bars represent 200 *μ*m. The TG was divided into V1, V2, and V3 regions indicated by three squares. Bottom panels are magnified sections of three squares in the upper panels; scale bars represent 20 *μ*m. (C) Quantification of TMJ primary afferent neurons in three sections of the TG. Dil-labeled TG neurons were distributed only in V3, but not V1 and V2; *n* = 6.

HE- and DAPI-stained longitudinal sections showed that TG included three subregions: V1 region (ophthalmic and anteromedial), V2 region (maxillary and anterolateral), and V3 region (mandibular and posterolateral; Figure 1A,B). Next, Dil was injected into the TMJ of rats to retrogradely label TG neurons projecting afferent fibers to the TMJ region. We found that Dil-labeled TG neurons were located only in V3, but not V1 and V2 (Figure 1B,C).

### 3.2. Upregulation of the Number of TG Neurons Innervating Rat TMJs During the Progression of TMJOA Chronic Pain

**Figure 2:**
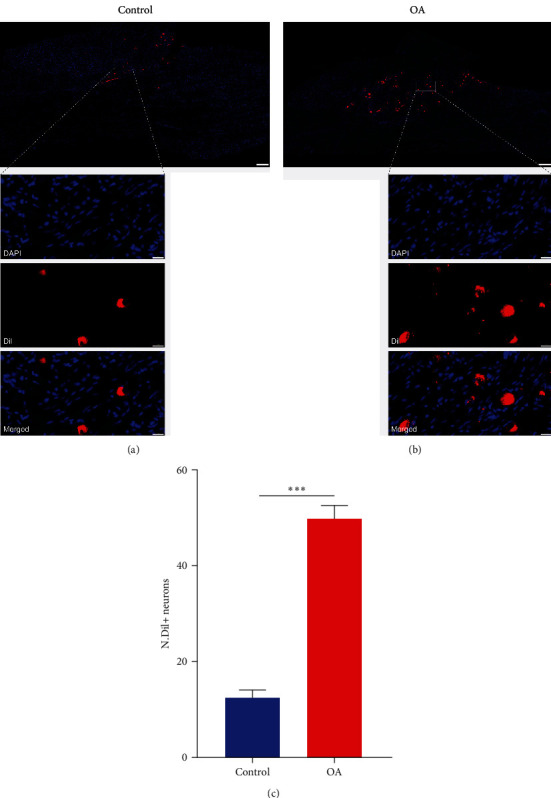
Upregulation of Dil+ TG neurons from rats of TMJOA chronic pain. (A,B) Representative immunofluorescent images of Dil-labeled (red) TG neurons at 14 days after saline (control) or MIA (OA) injection, respectively. DAPI (blue) was used for cell nuclei staining; scale bars represent 200 *μ*m. Bottom panels are magnified sections of squares in the upper panels; scale bars represent 20 *μ*m. (C) Quantitative analysis of the number of Dil+ neurons in TG;  ^*∗∗∗*^*P* < 0.001, two-tailed Student's *t*-test; *n* = 6. Data are shown as means ± standard deviations.

To further determine whether chronic pain in TMJOA influences the localization of TMJ primary afferent neurons in the TG, we established a TMJOA chronic pain animal model based on a recent study [17]. As in control rats, Dil-positive (Dil+) neurons remained located only in the V3 region of the TG in TMJOA rats, indicating that TMJOA chronic pain did not change the distribution of TMJ primary afferent neurons in rat TGs (Figure 2A,B). Interestingly, the number of Dil+ TG neurons was significantly higher in MIA-induced TMJOA rats than in the control group (Figure 2A–C;  ^*∗∗∗*^*P* < 0.001). Taken together, although there was a marked increase in the numbers of TMJ primary afferent neurons in rats after MIA injection, we did not observe a change in the spatial distribution of TMJ primary afferent neurons compared to saline-injected rats.

### 3.3. Small- and Medium-Sized Dil+ Neurons Instead of Large-Sized Dil+ Neurons in TGs Contribute to Chronic Pain in TMJOA Rats

**Figure 3:**
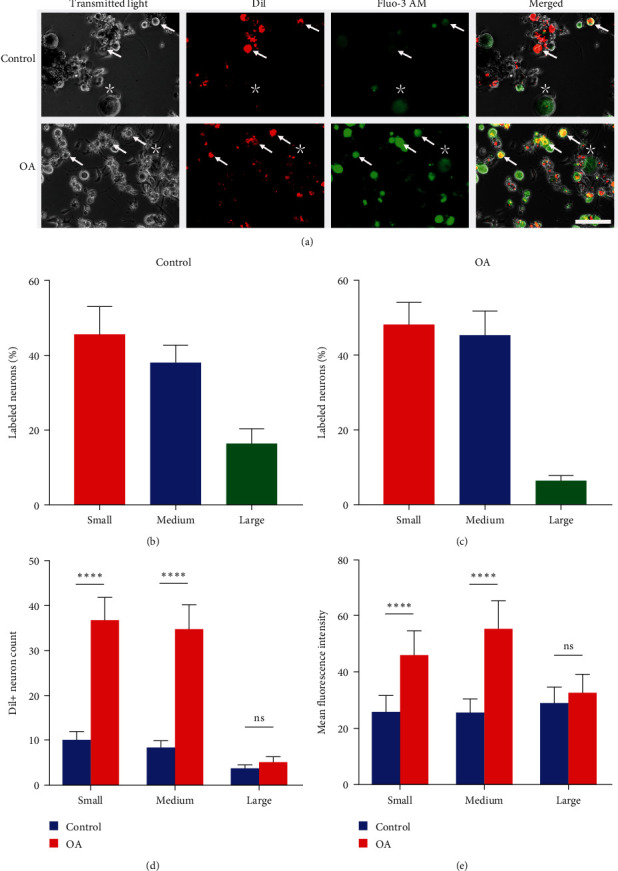
Activation of small- and medium-sized Dil+ TG neurons in TMJOA rats. (A) Representative images of calcium imaging of primary TG neurons in control rats or TMJOA rats; scale bars represent 100 *μ*m. Asterisks and arrowheads indicate the Dil-negative (Dil−) neurons and Dil-positive (Dil+) neurons, respectively. (B) Quantification of small-, medium-, and large-sized TMJ primary afferent neurons in TG from saline-treated rats; *n* = 6. (C) Quantification of small-, medium-, and large-sized TMJ primary afferent neurons in TG from MIA-treated rats; *n* = 6. (D) Quantitative analysis of the number of small- ( ^*∗∗∗∗*^*P* < 0.0001), medium- ( ^*∗∗∗∗*^*P* < 0.0001), and large-sized (ns, *P* > 0.05) Dil+ TG neurons, compared with the control group, two-tailed Student's *t*-test; *n* = 6. (E) Quantitative analysis of the mean fluorescence intensity of small- ( ^*∗∗∗∗*^*P* < 0.0001; Mann–Whitney's *U* test), medium- ( ^*∗∗∗∗*^*P* < 0.0001; two-tailed Student's *t*-test), and large-sized (ns, *P* > 0.05; two-tailed Student's *t*-test) Dil+ TG neurons, compared with the control group; *n* = 22–46 neurons/four rats. Data are shown as means ± standard deviations.

To identify the specific type of neurons that responded to TMJOA chronic pain, we analyzed the morphological changes of TG neurons innervating rat TMJs throughout the development of chronic pain in TMJOA. Before TMJOA, 45% (60/132), 38% (50/132), and 17% (22/132) of Dil+ neurons in rat TGs were small-, medium-, and large-sized neurons, respectively (Figure 3A,B). In TMJOA rats, small-, medium-, and large-sized neurons accounted for 48% (222/461), 45% (209/461), and 7% (30/461) of Dil+ TG neurons, respectively (Figure 3A,C), suggesting that TMJ-projecting TG neurons are predominantly composed of small- and medium-sized cells. Importantly, we also found that the number of small- and medium-sized Dil+ TG neurons in MIA-injected rats was significantly higher than that in the control group ( ^*∗∗∗∗*^*P* < 0.0001), while the number of large-sized neurons remained unchanged (Figure 3A,D; *P* > 0.05). To further examine whether the increased number of Dil+ TG neurons after MIA injection was responsible for TMJOA chronic pain, the calcium indicator Fluo-3 AM was loaded into the TG neurons to detect the intracellular calcium concentration ([Ca2+]i). Similarly, calcium imaging showed that the MFI of small- and medium-sized Dil+ TG neurons increased significantly in MIA-induced TMJOA rats relative to saline-injected rats ( ^*∗∗∗∗*^*P* < 0.0001), indicating increased [Ca2+]i, but the MFI of large-sized neurons remained constant (Figure 3A,E; *P* > 0.05). Taken together, these findings suggest that small- and medium-sized Dil+ TG neurons were activated to mediate TMJOA chronic pain induced by MIA.

### 3.4. Sensitization of CGRP + Dil+ and IB4 + Dil+ Neurons in Rat TGs Correlates With Chronic Pain in TMJOA

**Figure 4:**
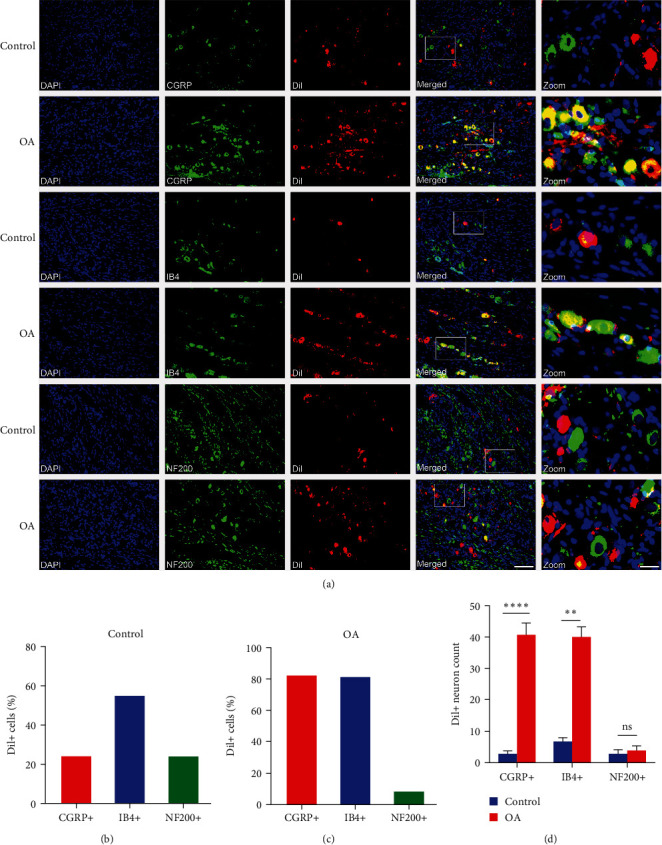
Sensitization of CGRP + Dil+ and IB4+Dil+ TG neurons from TMJOA rats. (A) Representative images of immunofluorescent analysis of Dil+ (red), CGRP+, IB4+, or NF200+ (green), and co-localization (yellow); DAPI (blue) was used for cell nuclei staining; scale bars represent 100 *μ*m. Zoomed panels are magnified immunofluorescent images; scale bars represent 25 *μ*m. (B) Quantification of CGRP+, IB4+, and NF200+ TMJ primary afferent neurons in TG from saline-treated rats; *n* = 6. (C) Quantification of CGRP+, IB4+, and NF200+ TMJ primary afferent neurons in TG from MIA-treated rats; *n* = 6. (D) Quantitative analysis of the number of CGRP + Dil+ ( ^*∗∗∗∗*^*P* < 0.0001), IB4 + Dil+ ( ^*∗∗*^*P* < 0.01), and NF200 + Dil+ (ns, *P* > 0.05) TG neurons, two-tailed Student's *t*-test; *n* = 6. Data are shown as means ± standard deviations.

Beyond the morphological classification described above, TMJ primary afferent neurons in the TG can also be divided into different subtypes based on their neurochemical characteristics. Immunofluorescence analysis of the expression of neurochemical markers (CGRP, IB4, and NF200) was generally used to assess peripheral nociceptive sensitization [11]. Based on a recent study [17], we further examined the neurochemical properties of primary afferent neurons innervating the TMJ in rat TGs. In saline-treated rats, most TG neurons supplying the TMJ bound to IB4 (55%, *n* = 41/75), followed by CGRP (24%, *n* = 18/75), and NF200 (24%, *n* = 18/75) (Figure 4A,B). In MIA-treated rats, TG neurons supplying TMJ predominantly were bound to IB4 (81%, *n* = 240/298) and CGRP (82%, *n* = 244/298) and bound less to NF200 (8%, *n* = 24/298; Figure 4A,C). Importantly, the number of CGRP + Dil+ and IB4 + Dil+ TG neurons was significantly increased in MIA-treated rats compared with the control group, while the number of NF200 + Dil+ neurons remained the same (Figure 4A,D). Collectively, these results indicate that the sensitization of CGRP+ and IB4+ neurons labeled with Dil in the TG potentially contributes to TMJOA chronic pain in rats.

## 4. Discussion

The diagnosis and treatment of TMJOA pain are challenging work because of the limited knowledge about the mechanism. TG neurons are critical for transmitting noxious stimuli from the TMJ to the central nervous system and play a key role in the production of TMJOA pain [7, 20]. Changes in morphological and neurochemical properties of primary sensory neurons are related to the generation and maintenance of pain [6, 7]. Here, we found the morphological and neurochemical phenotype alterations of TG neurons innervating the TMJ in MIA-induced TMJOA chronic pain rats. Our findings suggest that increased numbers of TG neurons participate in sensory innervation of the TMJ in MIA-injected rats compared to saline-injected rats. Furthermore, recruitment and activation of small- and medium-sized Dil+ neurons and sensitization of CGRP- and IB4-positive Dil+ neurons in TG are involved in TMJOA chronic pain.

The TMJ is mainly innervated by the mandibular branches of the trigeminal ganglia, and the skin area over the TMJ is innervated by both maxillary and mandibular branches of the trigeminal ganglia [21, 22]. Indeed, our retrograde tracing data demonstrated that Dil-labeled TMJ primary afferent neurons were located only in the V3 region of the TG. This finding is in accordance with previous studies in rats [21]. One study demonstrated that the tracer did not permeate the intra-articular space even in rats with OA [23]; yet, another study showed that the tracer had leaked from the joint into the surrounding tissues [24]. Technical factors likely contribute to the differences in experimental results due to the high level of manual and technical skills for this particular intra-articular injection. The consistent finding of no labeled afferents in the V1 and V2 regions of the TG even in rats with TMJOA further supported the specificity of this intra-TMJ injection and retrograde labeling.

At present, research on the causes of chronic pain in TMJOA has mainly focused on sensory innervation in degenerative TMJ. Several studies have reported that an elevated level of local sensory innervation is a key step in the pathogenesis of pain associated with bone disease [13, 25]. Consistent with previous reports, we observed that the total number of Dil+ TG neurons have significantly increased in TMJOA rats induced by MIA, suggesting that overall sensory nerve innervation in the TMJ was increased in MIA-induced TMJOA rats. This increase may be attributable to an elevated level of sensory innervation in the condylar subchondral bone. Because previous studies have shown that knee osteoarthritis (KOA) may promote nerve growth in the subchondral bone, which could lead to disease progression and pain [25, 26]. In addition, a recent study on spinal pain caused by lumbar instability has reported that elevated levels of sensory innervation of the sclerotic endplate are the main cause of low back pain [13].

Morphological studies of nociceptors demonstrate the heterogeneity in the function of different subsets of primary sensory neurons [8, 14]. In general, small- to medium-sized neurons transmit nociceptive information, whereas large neurons transmit proprioceptive information [8]. Indeed, the number of small- and medium-diameter Dil+ TG neurons significantly has increased in MIA-injected rats compared with control rats, while the number of large-sized neurons remained constant. Consistent with our results, previous studies have revealed that the number of small- to medium-sized neurons continuously increases in response to anterior cruciate ligament ransection (ACLT)-induced knee pain [25]. Calcium ions are essential for neuronal activation; elevated [Ca2+]i is generally considered to reflect the increased excitability of neurons, as [Ca2+]i has often been used as a marker of neuronal excitability [27–29]. We recently reported that [Ca2+]i in TG neurons significantly has increased in MIA-induced chronic TMJOA pain [19]. In the current study, we further defined the specific type of neurons that responded to TMJOA chronic pain by calcium imaging and found an increase in [Ca2+]i in small- and medium-sized Dil+ TG neurons from rats with TMJOA chronic pain. Our results are consistent with in vivo calcium imaging studies, which show an increased percentage of responses in small- and medium-sized neurons in DRG and an increase in [Ca2+]i following stimulation in destabilization of the medial meniscus (DMM) mice compared to sham-operated mice [28]. Furthermore, the fact that we found activation of small- to medium-sized TG neurons is consistent with an electrophysiological study conducted in a rat model of KOA pain [30]. Specifically, the firing rate of small- to medium-sized DRG neurons increases on Day 14 after MIA injection, in response to KOA pain. Thus, our findings suggest that small- and medium-sized Dil+ TG neurons become activated by 14 days after MIA injection, as opposed to larger-sized neurons being recruited.

We were particularly interested in the neurochemical signatures of sensory neurons in the TG innervating the TMJ, as several studies have reported correlations between the size of sensory neuron cell bodies and their specific neurochemistries [31, 32]. Specifically, small- and medium-diameter peripheral sensory neurons are classified as peptidergic neurons that are CGRP-positive or as nonpeptidergic neurons, which are IB4-positive. In contrast, large-diameter sensory neurons are marked with NF200. Our retrograde labeling data showed that the number of CGRP + Dil+ and IB4 + Dil+ neurons in the TG was significantly increased in TMJOA chronic pain rat compared to control rats, while the number of Dil-labeled NF200+ neurons remained constant. These results indicate that sensitization of IB4 + Dil+ and CGRP + Dil+ neurons in rat TGs may be involved in TMJOA chronic pain. Consistent with our findings, a recent study has shown that different neuronal populations may participate in mechanical hypersensitivity induced in different pain models, such as IB4+ sensory neurons which are involved in the mechanical hypersensitivity of the inflammatory pain model but not in the spared peripheral nerve injury-induced neuropathic pain model [9]. Another study found that the number of CGRP+ nociceptors and the density of CGRP+nociceptive nerve endings significantly increased in the subchondral bone of KOA pain animals [25]. Accordingly, the way in which these CGRP+ and IB4+ nociceptors innervate the subchondral bone over the TMJOA progression remains to be investigated.

There are some limitations in this study. For detecting calcium concentrations, we used an in vitro method that may not ideally mimic the complex environment of a living animal; more advanced in vivo calcium imaging techniques are those which allow the detection of calcium concentrations within a living animal and provide more precise, real-time recordings of calcium signaling. In addition, future studies incorporating an anterograde tracing approach would further complement our current research. The MIA-induced TMJOA rat model mimics TMJOA pathology in humans in various aspects, such as cartilage degradation, subchondral bone changes, synovitis, and pain behavior. However, the MIA-induced TMJOA rat model cannot mimic the complex etiology of human TMJOA, such as genetic predisposition and mechanical and environment factors.

## 5. Conclusion

Collectively, our data suggest that sensitization of small- to medium-sized Dil+ neurons and CGRP- and IB4-positive Dil+ neurons in TGs may contribute to the development of chronic pain in the MIA-induced TMJOA rat model. This not only helps to elucidate the neurologic changes of TMJOA chronic pain but also provides new insights for treating TMJOA chronic pain.

## Data Availability

The raw data supporting the conclusions of this article will be available by the authors, without undue reservation.
